# Association Between Platelet Levels on Admission and 90-day Mortality in Patients With Exertional Heatstroke, a 10 Years Cohort Study

**DOI:** 10.3389/fmed.2021.716058

**Published:** 2021-11-11

**Authors:** Li Zhong, Ming Wu, Jingjing Ji, Conglin Wang, Zhifeng Liu

**Affiliations:** ^1^Department of Critical Care Medicine, The First Affiliated Hospital, Guizhou University of Chinese Medicine, Guiyang, China; ^2^Department of Critical Care Medicine and Infection Prevention and Control, The Second People‘s Hospital of Shenzhen & First Affiliated Hospital of Shenzhen University, Health Science Center, Shenzhen, China; ^3^Department of Critical Care Medicine, General Hospital of Southern Theater Command of PLA, Guangzhou, China; ^4^The First School of Clinical Medicine, Southern Medical University, Guangzhou, China

**Keywords:** heatstroke, platelets, APACHE II scores, SOFA scores, mortality

## Abstract

**Background:** Heatstroke is a common clinical symptom in summer with high mortality requiring identification of appropriate and rapid methods of assessment.

**Method:** This is a retrospective study that included the recent 10 years clinical data of heatstroke patients. A total of *n* = 186 patients were included in this study and grouped based on platelet (PLT) abnormality observed on admission.

**Results:** In the study group, *n* = 120 patients (64.5%) patients had normal PLT and *n* = 66 patients (35.5%) had abnormal PLT. Compared with PLT-normal group, PLT-abnormal group had higher Acute Physiology and Chronic Health Evaluation II (APACHE II) scores [median 15.0 (IQR 11.5–21.5) vs. 9.0 (IQR 7.0–12.5)] and SOFA scores [median 6.0 (IQR 4.0–10.0) vs. 2.0 (IQR 2.0–4.0)], lower Sequential Organ Failure Assessment (GCS)[median 8.0 (IQR 5.0–12.0) vs. 13.0 (IQR 9.0–14.0)]. The PLT-abnormal group had severe organ damage, including damage to the coagulation system, liver, and kidney (all *p* < 0.05). Significant differences were noted in 90-day survival between the two groups even after correction for Age, GCS, White blood cell count (WBC), Neutrophil, International normalized ratio (INR), Activated partial thromboplastin time (APTT), Procalcitonin (PCT), Alanine aminotransferase (ALT), Creatine (CR), D-Dime (D-D) (Before correction *P* < 0.001; After correction *P* = 0.009).The area under the ROC curve for the prediction of mortality based on PLT was 80.7% (95% CI 0.726–0.888, *P* < 0.001), the optimal cutoff value was 94, the sensitivity was 77.3%, and the specificity was 82.6%.

**Conclusion:** Patients with heatstroke with platelet abnormalities during admission have more severe organ impairment and a lower 90-day survival rate even when adjusted for other factors.

## Introduction

Heatstroke is a common clinical symptom in any season especially in summer, manifesting as multi-system inflammation caused by central hyperthermia and eventually multi-organ dysfunction, mortality up to 40% (1, 2). Heatstroke can be clarified in exertional heatstroke or classic heatstroke. Classic heatstroke occurs mostly in older patients, especially those with chronic diseases, they have weak ability to regulate thermal stimulation and are easily affected by high temperature stimulation. Exertional heatstroke usually occurs in athletes, soldiers or worker engaged in outdoor physical labor and it usually occurs under the condition of continuous high temperature and high intensity exercise. The best way to diagnose heatstroke is with rapid body temperature assessment and evaluation of central nervous system status. Since much of the research on exertional heatstroke indicates cellular damage occurs when body temperature exceeds 40.5 C for 30 min. It is very important to find an effective indicator to quickly evaluate the severity and prognosis of the disease. Platelets stop bleeding and participate in thrombosis (3). Platelet abnormality is a risk factor for evaluating the prognosis of a variety of diseases (4–6). Hence this study, retrospectively analyzed the clinical data of patients admitted to hospital for severe heatstroke in the past 10 years, and evaluated early platelet changes on the prognosis of patients with heatstroke for timely and effective clinical treatment.

## Methods

### Study Design and Participants

This single-center retrospective case-control study collected all patients with heatstroke admitted to the ICU of the General Hospital of Southern Theater Command in China from October 2008 to May 2019.

The inclusion criteria were as follows: (1) age > = 18 years old; (2) met the diagnostic criteria of severe heatstroke described below; (3) The diagnostic criteria for heatstroke were as follows: classic or exertional heatstroke with a history of exposure to hot and humid weather or strenuous activity, concurrent hyperthermia (central temperature above 40°C), and neurological dysfunction, including delirium, cognitive disorders, and disturbed consciousness. The exclusion criteria were as follows: (1) existing irreversible underlying diseases affecting mortality and (2) pregnancy or breastfeeding.

### Research Procedures

The base data of patients were collected, including inflammatory and organ function parameters on admission. Patients were divided into the PLT-normal and PLT-abnormal group according to whether their PLT is normal or not. The main result was 90-day mortality. Survival curve analysis was also performed and ROC curve analysis of PLT to assess the 90-day mortality of heatstroke patients. The PLT-abnormal group was further divided into the Survivors subgroup and Non-survivors subgroup, and the characteristics of the subgroups were compared.

### Definitions

Diagnostic criteria for platelet abnormalities: platelet count (PLT) <100(1 × 10^9^/L) or platelet count >300(1 × 10^9^/L).

### Statistical Analysis

The categorical data are expressed as numbers and percentages, and intergroup comparisons were performed with the Mann-Whitney U test, χ^2^ test, or Fisher's exact test. Continuous variables are expressed as the medians and interquartile ranges. Continuous data with a Gaussian distribution were compared with Student's *t*-test or one-way ANOVA, and those with a non-Gaussian distribution were compared with the Wilcoxon rank-sum test. The patient endpoint event was death within 90 days after onset. The survival curve was drawn using the Kaplan-Meier method. Significant indicators were identified using single-factor analysis, ROC curves were used to predict the prognosis of patients according to PLT. Statistical analyses were performed using the SPSS Windows version 11.0 statistical package (SPSS Inc, Chicago, IL), and *P* values (two-tailed) <0.05 were considered statistically significant.

## Results

### Demographics and Baseline Characteristics

**Figure 1:**
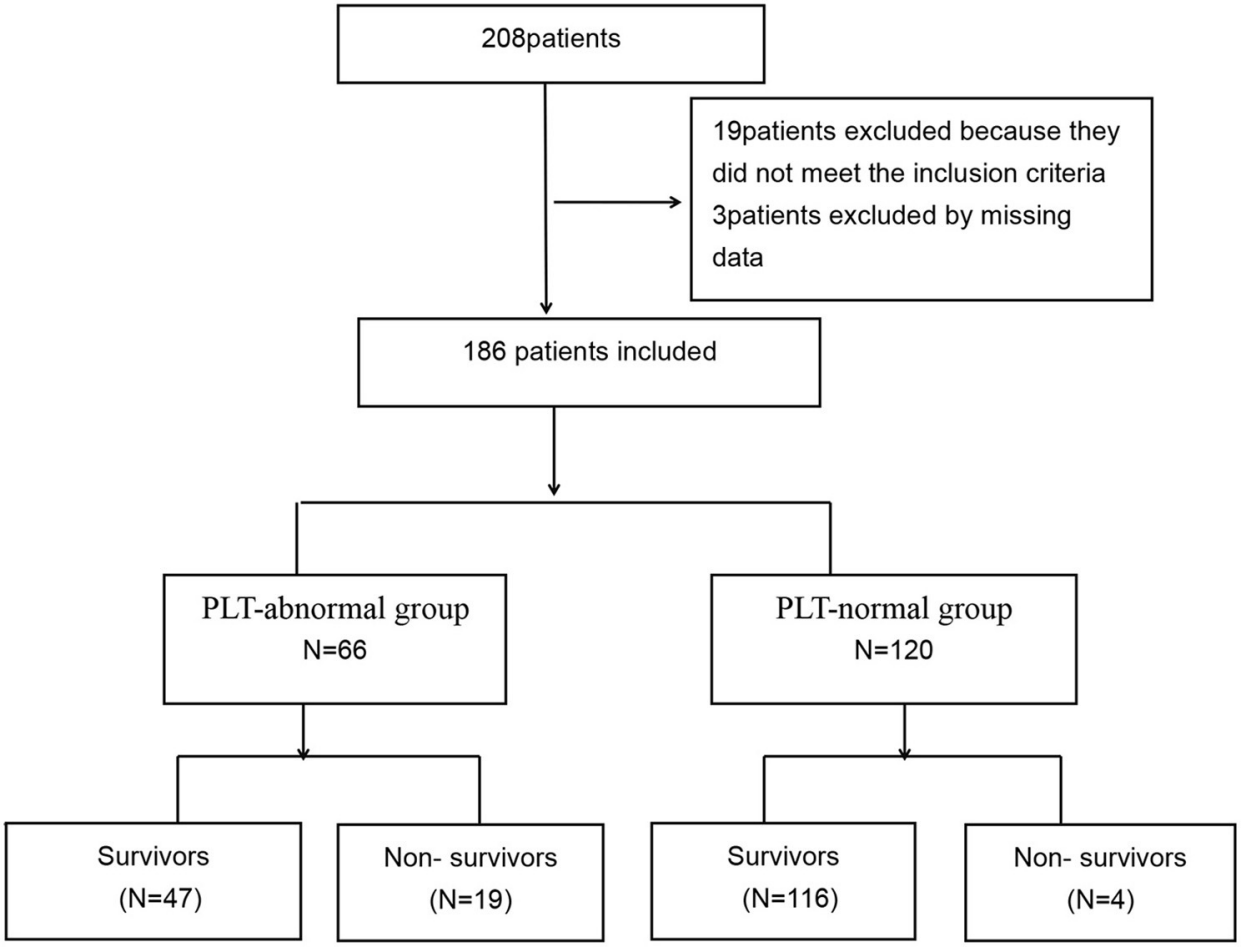
Flowchart of all excluded and included patients.

A total of 208 patients were included. After screening, 28 patients were excluded because they did not meet the inclusion criteria, 3 patients were excluded by missing data, 19 patients excluded because they did not meet the inclusion criteria, and 186 patients were finally included (Figure 1). All 186 patients were male, with a mean age of 21.0 years (IQR 19.0–27.0), Acute Physiology and Chronic Health Evaluation II (APACHE II) scores of 11.0 (IQR 8.0–16.0), Sequential Organ Failure Assessment (SOFA) scores of 3.0 (IQR 2.0–6.0), and GCS score of 12.0 (IQR 7.0–14.0). There were 120 patients (64.5%) patients with normal PLT and 66 patients (35.5%) with abnormal PLT.

### Comparison of Groups

Compared with PLT-normal group, PLT-abnormal group had higher APACHE II scores [median 15.0 (IQR 11.5–21.5) vs. 9.0 (IQR 7.0–12.5)] and SOFA scores [median 6.0 (IQR 4.0–10.0) vs. 2.0 (IQR 2.0–4.0)], lower GCS [median 8.0 (IQR 5.0–12.0) vs. 13.0 (IQR 9.0–14.0)]. PLT-abnormal group had severe organ damage, including damage to the coagulation system, liver and kidney (all *p* < 0.05) (Table 1).

**Table 1:** Baseline characteristics of clinical and laboratory findings in PLT-normal group and PLT-abnormal group.

	**Total**	**PLT-normal**	**PLT-abnormal**	***P* value**
	**(*N* = 186)**	**(*N* = 120)**	**(*N* = 66)**	
APACHII score, median (IQR)	11.0 (8.0–16.0)	9.0 (7.0–12.5)	15.0 (11.5–21.5)	<0.001
SOFA score, median (IQR)	3.0 (2.0–6.0)	2.0 (2.0–4.0)	6.0 (4.0–10.0)	<0.001
GCS score, median (IQR)	12.0 (7.0–14.0)	13.0 (9.0–14.0)	8.0 (5.0–12.0)	<0.001
Age (years), median (IQR)	21.0 (19.0–27.0)	20.0 (19.0–25.0)	23.0 (19.8–37.3)	0.003
WBC (1 × 10^9^/L), median (IQR)	11.4 (8.6–14.7)	11.7 (9.0–15.6)	10.1 (7.9–13.3)	0.020
Neutrophil (1 × 10^9^/L), median (IQR)	8.9 (6.5–12.5)	9.3 (6.6–13.2)	8.6 (6.5–10.9)	0.237
Lymphocyte (1 × 10^9^/L), median (IQR)	1.1 (0.6–1.9)	1.4 (0.8–2.2)	0.6(0.4–1.4)	<0.001
Monocytes (1 × 10^9^/L), median (IQR)	0.7 (0.4–1.0)	0.8 (0.4–1.1)	0.6 (0.3–0.7)	0.001
Platelets (1 × 10^9^/L), median (IQR)	165.0 (81.5–219.0)	187.0 (158.0–226.8)	62.0 (34.5–86.8)	<0.001
Mean platelet volume (%)	10.7 (10.1–11.5)	10.6 (10.1–11.1)	10.9 (10.2–11.7)	<0.001
Platelet distribution width (%)	12.5 (11.5–13.8)	12.0 (11.2–13.3)	13.5 (11.8–15.2)	<0.001
TBIL (μmol/L), median (IQR)	15.9 (10.1–29.5)	11.8 (8.7–18.8)	31.2 (16.1–65.3)	<0.001
ALT (U/L), median (IQR)	34.5 (20.0–228.8)	26.0 (17.0–47.0)	221.0 (53.8–1,606.3)	<0.001
AST (U/L), median (IQR)	66.5 (34.3–228.0)	44.0 (28.0–84.5)	290.0 (110.0–1,587.0)	<0.001
BUN (mmol/L), median (IQR)	5.8 (4.5–7.6)	5.6 (4.2–6.8)	6.4 (5.0–8.7)	0.009
CR (μmol/L), median (IQR)	127.5 (92.0–163.0)	125.0 (94.0–146.0)	147.5(87.8–223.5)	0.009
Cystatin C (mg/L)	1.0 (0.9–1.2)	1.0 (0.8–1.2)	1.1 (0.9–1.5)	0.003
CK (U/L), median (IQR)	912.0 (341.0–2,554.0)	585.5 (252.0–1,601.8)	1,688.5 (814.5–5,465.8)	<0.001
CKMB (ng/ml), median (IQR)	8.9(4.5–20.4)	5.7(3.8–14.8)	18.0(8.4–37.7)	<0.001
Mb (ng/ml), median (IQR)	468.9 (122.0–1,000.0)	265.4 (69.2–840.9)	789.0 (219.0–1,000.0)	0.001
CTNI (ng/ml), median (IQR)	0.11 (0.02–0.43)	0.06 (0.01–0.2)	0.28 (0.11–1.11)	<0.001
PT (s), median (IQR)	15.9 (14.1–20.6)	15.0 (13.9–16.3)	23.5 (16.6–37.4)	<0.001
INR median (IQR)	1.3 (1.1–1.8)	1.2 (1.1–1.3)	2.0 (1.3–3.7)	<0.001
APTT (s), median (IQR)	39.0 (33.5–49.5)	35.6 (32.2–40.9)	58.1 (41.4–99.9)	<0.001
TT(s), median (IQR)	17.6 (16.6–21.3)	17.2 (16.3–18.0)	22.6 (17.6–54.0)	<0.001
FIB (g/L), median (IQR)	2.5 (2.0–2.9)	2.5 (2.2–3.1)	2.2 (1.5–2.8)	0.003
D-D (mg/L), median (IQR)	1.7 (0.5–7.0)	0.8 (0.4–2.7)	6.6 (2.0–13.3)	<0.001
CRP (mg/dl), median (IQR)	3.3 (1.4–6.6)	3.2 (0.7–5.6)	3.4 (3.2–9.8)	0.016
PCT (ng/ml), median (IQR)	1.9 (0.8–4.3)	1.3 (0.6–3.3)	3.2 (1.4–5.9)	<0.001
RM	83/174(47.7%)	40 (48.2%)	43 (51.8%)	<0.001
AKI	80/185 (43.2%)	41 (51.2%)	39 (48.8%)	0.001
RM&AKI	40/181 (22.1%)	13 (32.5%)	27 (67.5%)	<0.001

*IQR, Inter-Quartile Range; APACHE II, Acute Physiology and Chronic Health Evaluation II score; SOFA, Sequential Organ Failure Assessment; GCS, Glasgow Coma Score; WBC, White blood cell count; TBIL, Total bilirubin; ALT, Alanine aminotransferase; AST, Aspartate aminotransferase; BUN, Blood urea nitrogen; CR, Creatine; CK, Creatine Kinase; CK-MB, Creatine kinase isoenzymes; CTNI, Cardiac troponin I; PT, Prothrombin time; INR, International normalized ratio; APTT, Activated partial thromboplastin time; TT, Thrombin time; FIB, Fibrinogen; D-D:D-Dimer; CPR, C-reactive protein; PCT, Procalcitonin*.

### Main Outcomes

**Figure 2:**
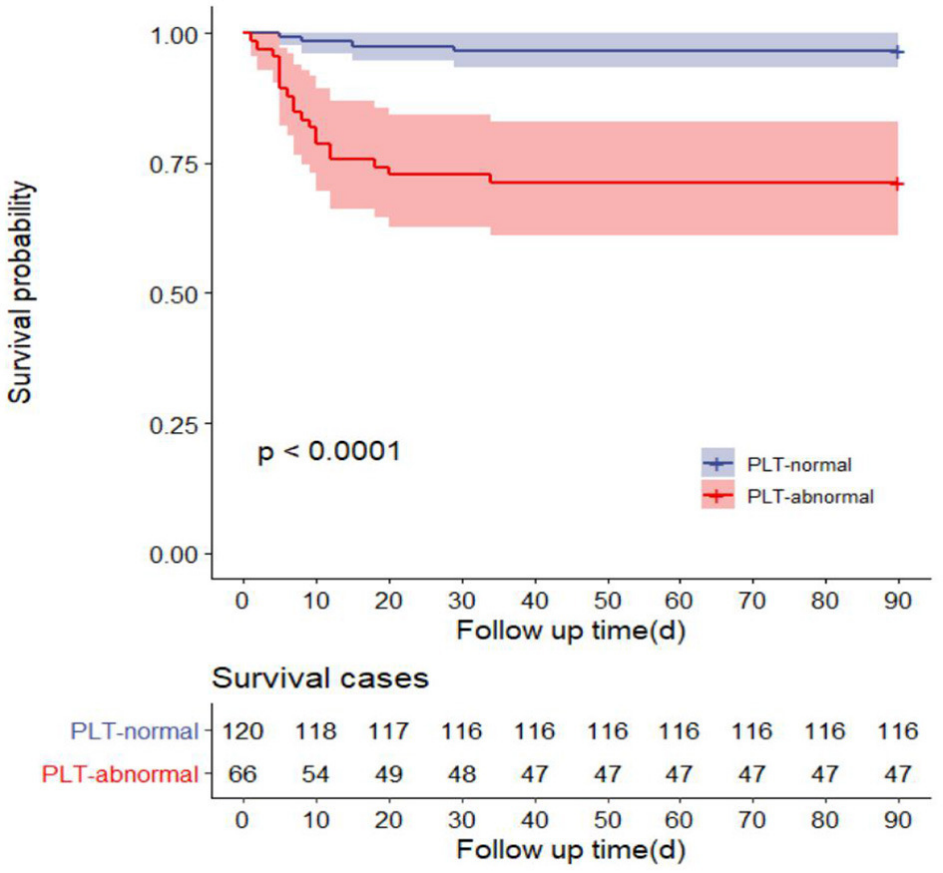
Survival curves of 90-day mortality rate in PLT-abnormal group and PLT-normal group.

The 90-day mortality rate of the PLT-abnormal group was 29% (19/66), while that of the PLT-normal group was 3.3% (4/120). There were statistically significant differences in 90-day survival between the two groups even after correction for Age, GCS, White blood cell count (WBC), Neutrophil, International normalized ratio (INR), Activated partial thromboplastin time (APTT), Procalcitonin (PCT), Alanine aminotransferase (ALT), Creatine (CR), D-Dime (D-D) (Before correction *P* < 0.001; After correction *P* = 0.009) (Table 2). Survival analysis showed that the 90-day survival time of patients with PLT-abnormal group was shorter than that of the PLT-normal group (*P* < 0.001) (Figure 2).

**Table 2:** Outcomes of PLT-normal group and PLT-abnormal group.

**All cohort**	**PLT-normal**	**PLT-abnormal**	***P*-value**	*P*-value^*^
	**(*n* = 120)**	**(*n* = 66)**		
90-day fatality			<0.001	0.009
Survivor	116(96.7%)	47(71%)		
Non-survivor	4(3.3%)	19(29%)		

* *Adjusted: Age, GCS, WBC, Neutrophil, INR, APTT, PCT, ALT, SCR, D-D*.

### Subgroup Analysis

In the subgroup analysis of the PLT-abnormal group, there were 47 survivors (71.2%) and 19 Non-survivors (28.8%).Compared with survivors, non-survivors had higher APACHE II scores [median 22.0 (IQR 17.8–23.5) vs. 14.0 (IQR 10.0–18.0)], higher SOFA scores [median 13.5 (IQR 8.8–15.0) vs. 6.0 (IQR 3.0–7.0)], lower GCS [median 4.0 (IQR 3.0–7.3) vs. 10.0 (IQR 6.0–14.0)]. In addition, kidney and blood coagulation system was more serious in the non-survivors (*P* < 0.05), (Table 3).

**Table 3:** Baseline characteristics of survivors and non-survivors in patients with PLT-abnormal.

	**Total**	**Survivors**	**Non-survivors**	***P* value**
	**(*N* = 66)**	**(*N* = 47)**	**(*N* = 19)**	
APACHII score, median (IQR)	15.0 (11.5–21.5)	14.0 (10.0–18.0)	22.0 (17.8–23.5)	0.001
SOFA score, median (IQR)	6.0 (4.0–10.0)	6.0 (3.0–7.0)	13.5 (8.8–15.0)	<0.001
GCS score, median (IQR)	8.0 (5.0–12.0)	10.0 (6.0–14.0)	4.0 (3.0–7.3)	0.002
Age (years), median (IQR)	23.0 (19.8–37.3)	26.0 (20.0–39.0)	22.0 (18.0–24.0)	0.077
WBC (1 × 10^9^/L), median (IQR)	10.1 (7.9–13.3)	10.1 (8.3–12.5)	9.8 (7.6–14.2)	0.927
Neutrophil (1 × 10^9^/L), median (IQR)	8.6 (6.5–10.9)	8.6 (6.8–10.9)	7.4 (5.9–11.8)	0.620
Lymphocyte (1 × 10^9^/L), median (IQR)	0.6 (0.4–1.4)	0.6 (0.4–1.1)	0.5 (0.3–3.1)	0.788
Monocytes (1 × 10^9^/L), median (IQR)	0.6 (0.3–0.7)	0.5 (0.3–0.7)	0.7 (0.2–0.8)	0.854
Platelets (1 × 10^9^/L), median (IQR)	62.0 (34.5–86.8)	59.0 (37.0–91.0)	71.0 (29.0–84.0)	0.600
Mean platelet volume (%)	11.2 (10.5–11.8)	11.2 (10.4–11.9)	11.1 (10.5–11.9)	0.837
Platelet distribution width (%)	13.3 (11.8–15.2)	13.3 (11.5–11.4)	13.2 (12.0–16.6)	0.430
TBIL (μmol/L), median (IQR)	31.2 (16.1–65.3)	27.6 (15.1–61.6)	37.4 (21.2–127.8)	0.183
ALT (U/L), median (IQR)	221.0 (53.8–1,606.3)	162.0 (44.0–1,314.0)	246.0 (87.0–1,897.0)	0.350
AST (U/L), median (IQR)	290.0 (110.0–1,587.0)	190.0 (81.0–160.0)	406.5 (112.5–2,270.0)	0.315
BUN (mmol/L), median (IQR)	6.4 (5.0–8.7)	5.8 (4.9–7.7)	8.1 (6.2–10.0)	0.058
CR (μmol/L), median (IQR)	147.5 (87.8–223.5)	109.0 (81.0–160.0)	228.0 (187.0–286.0)	<0.001
Cystatin C (mg/L)	1.1 (0.9–1.5)	1.0 (0.8–1.3)	1.5 (1.1–3.4)	0.001
CK (U/L), median (IQR)	1,688.5 (814.5–5,465.8)	1,796.5.0 (799.3–4,572.3)	1,672.0 (863.3–8,240.5)	0.500
CKMB (ng/ml), median (IQR)	18.0 (8.4–37.7)	16.4 (7.8–31.9)	19.9 (9.7–50.4)	0.299
Mb (ng/ml), median (IQR)	789.0 (219.0–1,000.0)	456.2 (124.3–1,000.0)	1,000.0 (935.1–1,000.0)	0.003
CTNI (ng/ml), median (IQR)	0.28 (0.11–1.11)	0.16 (0.02–0.46)	1.9 (0.90–3.86)	<0.001
PT (s), median (IQR)	23.5 (16.6–37.4)	20.3 (17.0–35.4)	37.5 (25.6–45.6)	0.003
INR median (IQR)	2.0 (1.3–3.7)	1.7 (1.3–2.5)	3.8 (2.4–5.0)	0.001
APTT (s), median (IQR)	58.1 (41.4–99.9)	46.3 (39.2–77.9)	91.2 (76.1–124.8)	0.003
TT (s), median (IQR)	22.6 (17.6–54.0)	20.3 (17.0–35.4)	38.7 (27.2–82.5)	0.004
FIB (g/L), median (IQR)	2.2 (1.5–2.8)	2.5 (1.9–2.8)	1.3 (0.9–2.1)	0.003
D-D (mg/L), median (IQR)	6.6 (2.0–13.3)	3.9 (0.9–11.4)	10.1 (9.8–20.0)	0.001
CRP (mg/dl), median (IQR)	3.4 (3.2–9.8)	3.4 (2.5–11.0)	3.4 (3.3–6.7)	0.886
PCT (ng/ml), median (IQR)	3.2 (1.4–5.9)	2.9 (1.2–5.4)	3.8 (1.7–8.8)	0.182

*IQR, Inter-Quartile Range; APACHE II, Acute Physiology and Chronic Health Evaluation II score; SOFA, Sequential Organ Failure Assessment; GCS, Glasgow Coma Score; WBC, White blood cell count; TBIL, Total bilirubin; ALT, Alanine aminotransferase; AST, Aspartate aminotransferase; BUN, Blood urea nitrogen; CR, Creatine; CK, Creatine Kinase; CK-MB, Creatine kinase isoenzymes; CTNI, Cardiac troponin I; PT, Prothrombin time; INR, International normalized ratio; APTT, Activated partial thromboplastin time; TT, Thrombin time; FIB, Fibrinogen; D-D, D-Dimer; CPR, C-reactive protein; PCT, Procalcitonin*.

### Risk Factor Analysis

**Figure 3:**
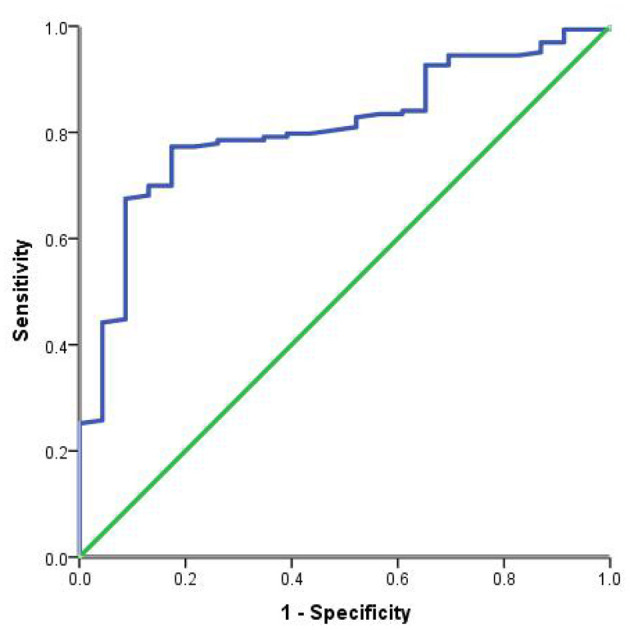
ROC curve analysis of PLT to assess the 90-day mortality of heat stroke patients.

The area under the ROC curve for the prediction of mortality based on PLT was 80.7% (95% CI 0.726–0.888, *P* < 0.001), the optimal cutoff value was 94 (1 × 10^9^/L), the sensitivity was 77.3%, and the specificity was 82.6% (Figure 3).

## Discussion

In this retrospective cohort study, patients with heat stroke and abnormal platelets at the time of admission had more serious organ function damage, mainly blood coagulation, liver, and kidney damage. Compared with patients without platelet abnormalities, patients with platelet abnormalities had a reduced 90-day survival rate. The cut-off value of PLT predicted by the ROC curve was 94(1 × 10^9^/L), the sensitivity was 77.3%, and the specificity was 82.6%.

In our study, it was found that 35.5% of heatstroke patients had abnormal platelets, of which thrombocytopenia was the majority. Those with platelet abnormalities will suffer more severe organ function damage, mainly blood clotting, liver, and kidney damage, which is consistent with previous studies (7). Platelet count and function play an important role in the coagulation lesions of heatstroke, and platelet activation is triggered by heat and/or endothelial injury (7). Heatstroke can lead to inflammation, coagulation, and direct cytotoxic effects, damage to endothelial cells, leading to microthrombosis, and eventually platelet depletion, which can lead to coagulation disorders and even DIC (8). Thrombocytopenia is an important finding in the development of HS-induced Acute kidney injury (AKI) (9, 10). AKI patients are combined with renal blood flow reduction, renal tissue ischemia, and hypoxia, fibrinolytic system disorders, causing abnormal coagulation, that are mainly manifested as platelet function loss (11). Heatstroke can lead to dysfunction of multiple organs, including liver damage. Serotonin from platelets are released and involved in pathophysiological processes such as liver injury response, regulation of liver function, and regeneration (12).

PLT has been evaluated in sepsis, diabetes, cardiovascular disease, and other diseases, and is correlated with disease severity and prognosis (13–16). PLT count is also part of the scoring for heatstroke patients (17). What makes our study different from other studies is that we investigated the effect of early platelet changes on 90-day prognosis in a population with exertional heat stroke, not limited to patients with AKI. In addition, we had a relatively long follow-up period, and prognostic parameters were adjusted for statistical analysis. The clinical characteristics of patients with platelet abnormality were analyzed in detail, which was not mentioned in other literatures. ROC curve analysis of platelets, and prediction of cut-off values, which further clarified the prognostic value of platelets for heatstroke patients. In our study, it was also found that compared with patients without platelet abnormality, the 90-day survival rate of patients with platelet abnormality was lower, and the sensitivity and specificity were 77.3% and 82.6% when the PLT of 94 (1 × 10^9^/L) was taken as the critical point for predicting the 90-day death of patients with severe heatstroke. In the pathophysiological process of heatstroke, the relationship between heatstroke and platelets is very complex. Heatstroke damages platelets through heat stress and inflammation, and platelets aggravate the condition by activating coagulation response and further amplifying inflammation response (18). Therefore, platelets can assess the severity of heatstroke patients to a certain extent, contribute to the evaluation of the prognosis of heatstroke patients, and provide a reference for the selection of clinical treatment options.

This study has some limitations. First, it is a single-center cohort study with a relatively small number of cases. A multi-center study will be conducted in the later stage to increase the sample size and increase the statistical reliability. Second, exertional heatstroke is a special disease that occurs mainly in people who are engaged in heavy physical labor and exercise, often in a predominantly male group. In addition, due to estrogen protection and other factors, women under equal conditions rarely develop heatstroke. Therefore, our study subjects are also mainly male and may not fully reflect the changes of platelets in the overall population. Third, it is true that early recognition and treatment is crucial for heatstroke survivability, and the patients in our study usually received basic treatment before admission, including simple cooling and symptomatic support therapy. However, these information were pre hospital data. This study is a retrospective study, so we can not accurately obtain all these information, which is also one of the limitations of this study.

## Conclusion

Patients with heatstroke who are admitted to hospital with platelet abnormalities have more severe organ impairment and a lower 90-day survival rate even when adjusted for other factors.

## Data Availability Statement

The original contributions presented in the study are included in the article/supplementary material, further inquiries can be directed to the corresponding author.

## Ethics Statement

The studies involving human participants were reviewed and approved by Medical Ethics Committee of the Southern Theater General Hospital. Written informed consent for participation was not required for this study in accordance with the national legislation and the institutional requirements.

## Author Contributions

LZ and MW: data collection and analysis and manuscript preparation. JJ and CW: data collection and analysis. ZL: study designed, data collection and analysis, manuscript preparation, and review. All authors read and approved the final manuscript.

## Funding

This work was supported by grants from the National Natural Science Foundation of China [NO. 82072143], grants from the Natural Science Foundation of Guangdong Province of China [NO. 2021A1515010170], and grants from the PLA Logistics Research Project of China [18CXZ030 and BLJ20J006].

## Conflict of Interest

The authors declare that the research was conducted in the absence of any commercial or financial relationships that could be construed as a potential conflict of interest.

## Publisher's Note

All claims expressed in this article are solely those of the authors and do not necessarily represent those of their affiliated organizations, or those of the publisher, the editors and the reviewers. Any product that may be evaluated in this article, or claim that may be made by its manufacturer, is not guaranteed or endorsed by the publisher.
